# Type III Congenital Pulmonary Airway Malformation: A Case Report

**DOI:** 10.30699/ijp.2024.2025527.3280

**Published:** 2025-08-01

**Authors:** Maryam Soltan, Farzaneh Nayeri

**Affiliations:** 1 *Department of Pathology, Assistant professor, Isfahan University of Medical Sciences, Isfahan, Iran *; 2 *Department of Pathology, Resident of pathology, Isfahan University of Medical Sciences, Isfahan, Iran*

**Keywords:** Congenital, Respiratory Distress, Cystic Adenomatoid Malformation of Lung, Newborn

## Abstract

Congenital pulmonary airway malformation (CPAM) is a structural anomaly that occurs during development of the lower respiratory tract. We describe a 4-day-old male infant with this uncommon congenital anomaly. He presented with respiratory distress and low oxygen saturation. A chest radiograph showed infiltration in the right lower lobe, and a chest computed tomography (CT) revealed alveolar opacity with an air bronchogram pattern in the right lung along with mediastinal shift. The right lower lobe was surgically resected. Pathological examination showed an 8-cm, predominantly solid cut surface with a rare tiny cyst, consistent with a congenital cystic adenomatoid malformation (type 3). Congenital pulmonary airway malformations are the most common congenital parenchymal lung anomalies. Although their development is debated, it is believed to result from a halt in fetal bronchial tree growth between the sixth and seventh weeks of fetal life. Flaws in thyroid transcription factor 1 have also been proposed. With the widespread use of high-quality ultrasonography in modern obstetrics, it is now less likely for congenital pulmonary airway anomalies to remain undetected until adulthood. Early surgical excision is generally recommended. However, in asymptomatic infants, management remains controversial because either operative or non-operative approaches may be used later in life, particularly in light of complications such as the potential for mucinous adenocarcinoma with a lepidic-predominant pattern. Patients with this condition in neonatal intensive care units should be managed by a multidisciplinary team that includes pediatric surgeons, neonatologists, and radiologists.

## Introduction

Congenital parenchymal lung malformations are rare but well-characterized fetal anomalies that typically present in children. They encompass a spectrum of defects, including congenital lobar hyperinflation, bronchogenic cysts, congenital cystic adenomatoid malformation (CCAM), and lobar sequestration (1).

Here, we describe a 4-day-old male who presented with respiratory distress due to CCAM and review the literature on the clinical manifestation of this congenital lung abnormality.

## Case Report

A male infant with respiratory distress and decreased oxygen saturation since birth was admitted to a referral pediatric hospital. Prenatal history and fetal ultrasound were both normal. He was delivered via cesarean section at 37 weeks of gestation, with a birth weight of 2730 g. His APGAR scores were 7 at one minute and 9 at five minutes.

Due to ongoing respiratory distress (respiratory rate of 150 breaths/min and SpO2 of 90%), a chest X-ray was performed at the birth hospital, which showed a mass (the original X-ray is not available at our facility). On admission to our hospital, arterial blood gas (ABG) analysis showed a pH of 7.42, pCO₂ of 36 mmHg, and HCO₃ of 23.4 mEq/L.

**Fig. 1:**
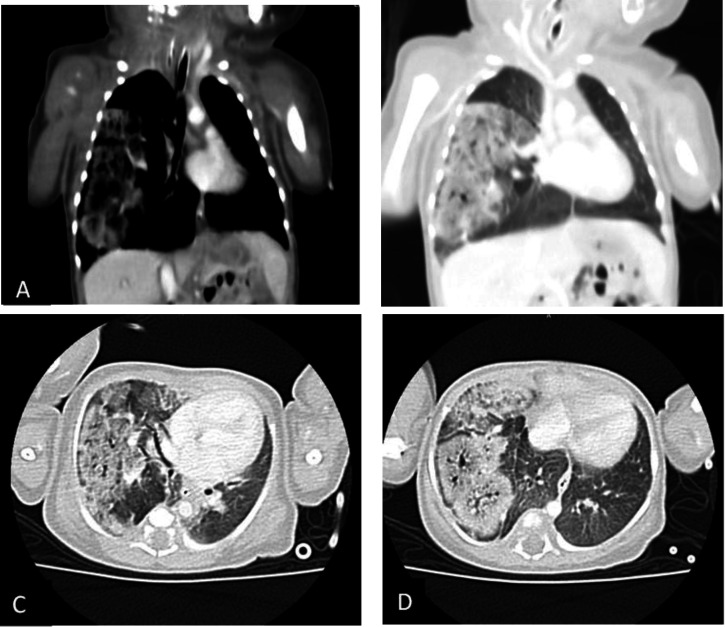
Coronal mediastinal window(A), coronal lung window(B), and axial lung windows (C and D) reveal the near entire right lower and right middle lobes ground glass opacity with multiple low-density areas (cystic change)

Physical examination revealed a scaphoid abdomen, and the infant was on mechanical ventilation. A chest computed tomography scan demonstrated alveolar opacity with an air bronchogram pattern in the right lung and a mediastinal shift. Diaphragmatic fluoroscopy was used to assess the integrity of the diaphragm (Figure 1).

**Fig. 2:**
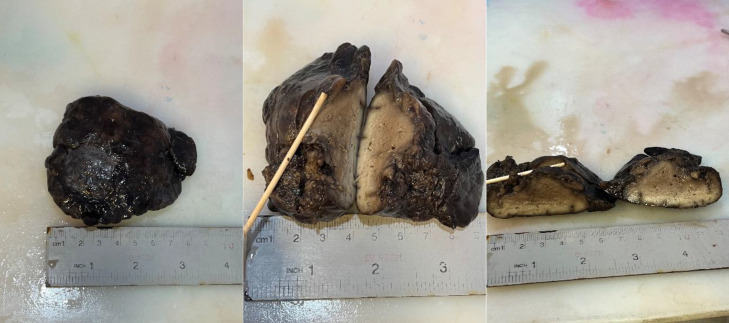
Macroscopic pictures

The patient underwent a thoracotomy and right lower lobectomy at 4 days of age due to ongoing respiratory distress and concern about potential complications.

Postoperatively, the patient had a difficult recovery because of pneumonia caused by multi-resistant *Klebsiella pneumoniae*, leading to respiratory distress and sepsis during hospitalization. This required an extended stay in the ICU for 100 days and necessitated a thoracostomy. Ultimately, after 115 days, the patient was discharged with parental consent.

**Fig. 3:**
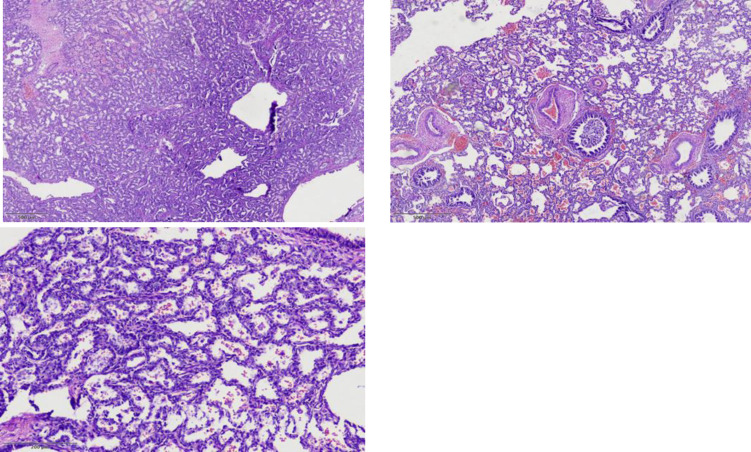
Randomly distributed irregular bronchiole-like structures, separated by alveolus-like structures, lined by cuboidal epithelial cells imparting an adenomatoid (or gland-like) appearance.

Pathological examination of the specimen revealed an 8-cm, predominantly solid congenital cystic adenomatoid malformation (CCAM), with a rare tiny cyst (Figure 2). Hematoxylin-eosin (H&E) staining showed small, irregular cystic cavities lined mainly by ciliated cuboidal to columnar epithelium. Some small cysts had thicker septa with enlarged mesenchyme and cuboidal or columnar epithelium (Figure 3).

**Table 1:** Expanded Stocker Classification for CPAM

	Type0	Type1	Type2	Type3	Type4
Frequency (%)	1-3	>65	20-25	8	2-4
Cyst size (max cm)	0.5	10	2.5	1.5	7
Epithelial lining	Ciliated Pseudostratified, tall columnar with goblet cells	Ciliated Pseudostratified, tall columnar	Ciliated, cuboidal, or columnar	Ciliated cuboidal	Flattened alveolar lining cells
Muscular wall thickness (micro m)	100-500	100-300	50-100	0-50	25-100
Mucous cells	Present in all cases	Present in ~33%	Absent	Absent	Absent
Cartilage	Present in all cases	Present in 5-10%	Absent	Absent	Rare
Skeletal muscle	Absent	Absent	Present in ~5%	Absent	Absent

## Discussion

Congenital pulmonary airway malformations (CPAMs) encompass a range of cystic and non-cystic lung lesions that arise from early airway maldevelopment, resulting from adenomatous hyperplasia in the respiratory tract epithelium. Their incidence varies from 1:10,000 to 1:35,000 live births (1–5).

Possible differential diagnoses include congenital diaphragmatic hernia, pulmonary sequestration, bronchogenic cysts, and congenital lobar emphysema. Historically, congenital cystic adenomatoid malformations (CCAMs) were classified according to Stocker types 1, 2, and 3 (6).

“Congenital pulmonary airway malformation (CPAM)” is a more recent term that better reflects the fact that not all of these lesions are cystic (Table 1) (7). Similar to our patient, the lesion is classified as type III, which is non-cystic.

The progression of developmental defects along the tracheobronchial tree—from major airways to bronchioles and alveoli—is represented by CPAM types 0 through 4. With the increased use of ultrasound in modern obstetric practice, CPAMs are often detected during routine prenatal care; children with this condition typically present with respiratory distress and recurrent pulmonary infections (8). In our patient’s case, he presented with respiratory distress at birth and was immediately referred to a pediatric referral hospital for urgent evaluation and management.

Fetal lesions may increase or remain stable in size and can be associated with polyhydramnios or hydrops fetalis, which portend a poor prognosis (9,10). However, our patient had a normal prenatal history.

This condition may also be associated with other congenital malformations such as anasarca, renal agenesis, Potter’s syndrome, pectus excavatum, or bile duct hypoplasia, but fortunately, our patient did not exhibit any of these (11).

Surgical excision is considered the best approach to confirm diagnosis and reduce the risk of pulmonary infections and malignancy (12). There are reports of CPAM-related malignant transformations into bronchioloalveolar carcinoma, rhabdomyosarcoma, and pleuropulmonary blastoma (13). Most cases of CPAM-associated bronchioloalveolar carcinoma occur in type 1 CPAM (14).

Whether to operate on small, asymptomatic lesions should be discussed on a case-by-case basis with the parents. Despite favorable long-term outcomes in most patients, regardless of whether surgical resection is performed, long-term complications, including cancer, have been reported in patients aged 30 to 40 years (15).
